# Stakeholder perspectives on the effectiveness of the Victorian Salt Reduction Partnership: a qualitative study

**DOI:** 10.1186/s40795-021-00414-6

**Published:** 2021-04-22

**Authors:** Emalie Rosewarne, Wai-Kwan Chislett, Briar McKenzie, Jenny Reimers, Kellie-Ann Jolly, Kirstan Corben, Kathy Trieu, Jacqui Webster

**Affiliations:** 1 The George Institute for Global Health, The University of New South Wales, Sydney, NSW 2006 Australia; 2 Victorian Health Promotion Foundation, Melbourne, VIC 3053 Australia; 3 National Heart Foundation of Australia, Melbourne, VIC 3008 Australia

**Keywords:** Salt reduction, Public health nutrition, Stakeholder perspectives, Population intervention

## Abstract

**Background:**

Interventions to reduce population salt intake are feasible and cost-effective. The Victorian Salt Reduction Partnership implemented a complex, multi-faceted salt reduction intervention between 2014 and 2020 in the Australian state of Victoria. This study aimed to understand stakeholder perspectives on the effectiveness of the Victorian Salt Reduction Partnership.

**Methods:**

Semi-structured interviews were conducted with Partnership and food industry stakeholders. The Consolidated Framework for Implementation Research was adapted for the Partnership intervention and used to guide the qualitative analysis.

**Results:**

Fourteen Partnership and seven food industry stakeholders were interviewed. The Partnership was viewed as essential for intervention planning and decision-making and an enabler for intervention delivery. The goals of capacity building and collaborative action were perceived to have been achieved. The implementation team executed intended intervention activities and outputs, with some adaptations to strategy. Barriers and enablers to implementation were identified by interviewees, such as compatibility of individual, organisational and Partnership values and building positive relationships between the Partnership and food industry, respectively. Legal, political, social, environmental, technological and economic factors affecting intervention design, delivery and outcomes were identified.

**Conclusions:**

Establishing a Partnership with diverse skills and experience facilitated collaborative action, capacity building and execution of the intervention. Monitoring and evaluating implementation informed strategy adaptations, which allowed optimisation of Partnership strategy. The importance of developing strong communication networks between strategic and implementation-levels was a key lesson.

## Background

Salt reduction interventions have been identified as feasible, cost-effective approaches to reduce the non-communicable disease burden attributable to excess salt consumption [1, 2]. Global estimates suggest salt intake is double the recommended daily maximum amount of 5 g per day [2, 3]. In 2017, diets high in salt resulted in almost 3.2 million deaths and more than 70 million disability adjusted life years (DALYs) globally [4]. High salt intakes cause high blood pressure, which was the attributable factor in more than 10 million global deaths and 381 million DALYs [4]. In an effort to reduce the salt-related non-communicable disease burden, in 2013, United Nations Member States committed to the global target of a 30% relative reduction in average population salt intake by 2025 [5].

**Fig. 1:**
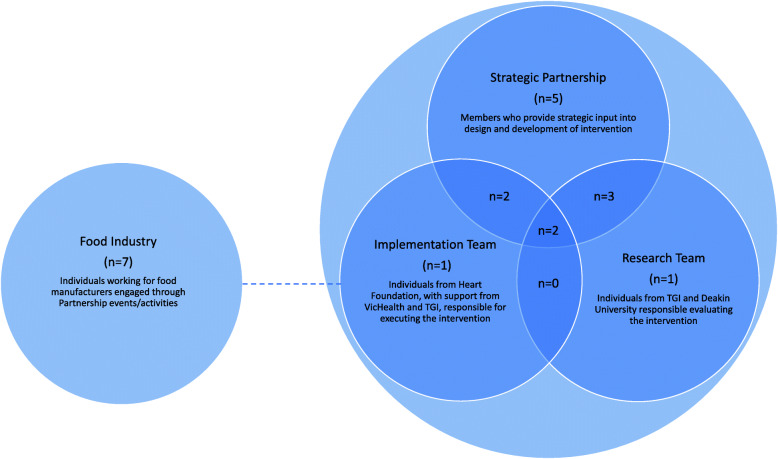
Structure, roles and responsibilities of the Partnership and food industry stakeholders. Broken line indicates engagement with Partnership through the intervention

Despite this commitment, coordinated efforts to reduce salt intake in many countries, including Australia, have been lacking [6, 7]. In the state of Victoria, salt intake was estimated at 8.9 g/day in adults [8] and 6.7 g/day in children [9], with men and boys consuming higher amounts than women and girls [8, 9] and both adults and children exceeding the recommended maximum salt intake [2]. To coordinate actions to reduce salt intake, in 2014 the Victorian Salt Reduction Partnership (referred to as the Partnership) was established by the Victorian Health Promotion Foundation (VicHealth) [10]. The Partnership, consisting of key organisations including VicHealth, The George Institute for Global Health (TGI), Heart Foundation, Deakin University Institute of Physical Activity and Nutrition (IPAN), and the Victorian Department of Health and Human Services (observer), developed a multi-component intervention strategy, informed by global evidence, to reduce the average salt intake of Victorians by 1 g per day by 2020 [10, 11]. The Partnership project, which is described in further detail elsewhere [12], comprised six main action areas, including four intervention arms (consumer awareness campaign, generate public debate, food industry engagement, and advocacy and policy strengthening), building a strong partnership and a research and evaluation component [12, 13]. The Partnership comprised the *Strategic Partnership* group, an implementation team and a research team (Fig. 1). The *Strategic Partnership* was tasked with providing strategic oversight to guide implementation of collaborative action and met quarterly throughout the intervention duration. The Heart Foundation was contracted by VicHealth to lead the delivery of the four intervention arms, with support from VicHealth and TGI through fortnightly meetings. Partnership organisations, led by TGI, secured NHMRC funding to conduct research and evaluate the intervention and met quarterly. Communication between Partnership groups was primarily through VicHealth.

To monitor and evaluate the effectiveness of the intervention, a comprehensive process and outcome evaluation was designed, as described in the protocol [12]. The aim of the process evaluation was to examine the reach, dose, fidelity, context, adoption and effectiveness of the intervention. This was done through the collection of routine administrative and cost data, impact assessments of consumer campaigns, product category reports, industry engagement, and advocacy activities, and analysis of stakeholder perspectives [12]. Semi-structured stakeholder interviews were conducted with key stakeholders at two timepoints: (1) in the early stages of intervention implementation (March to May 2017 [14]) and (2) towards the end of the intervention (May to December 2019). This paper reports on the interviews undertaken in 2019. The primary objective was to understand stakeholder perceptions of the effectiveness of the Partnership in achieving its intended role and delivering a salt reduction intervention. Key lessons and learnings generated from this research will be used with a view to contributing to greater understanding of how strategic partnerships can facilitate implementation of public health and nutrition interventions through collaborative action and capacity building.

## Methods

### Sample and recruitment

Potential participants were stakeholders involved in designing and/or delivering the Partnership intervention or engaged through the Partnership intervention, including the food industry, identified by VicHealth and the Heart Foundation. A letter of invitation to participate, along with the participant information sheet and consent form, was emailed to the VicHealth’s Partnership member contact list in April 2019, and Heart Foundation food industry contact list in August 2019. Participants agreed to participate by return email, including returning a signed consent form and agreeing to an interview time. Interviews were conducted from May to December 2019, either in person or online using Skype for Business or Zoom.

### Interviews

Semi-structured interviews were conducted by two researchers, E.R., a PhD candidate and dietitian at TGI who was not directly involved in the Partnership, and W-K.C., a research assistant at TGI who was not previously involved in the project. The survey instrument was previously developed for interim stakeholder interviews in 2017 to understand perceived barriers and enablers to intervention implementation [14] and was built on instruments used in similar studies [15, 16]. Questions centred on understanding the Partnership (effectiveness, structure and function), the process of executing the intervention, and internal and external factors affecting intervention design, delivery and outcomes. Questions were asked about each participant’s roles and their perceptions on delivery and fulfilment of their roles. The semi-structured approach allowed interviewers to adapt the questions based on each participant’s involvement in the Partnership, which enabled deeper knowledge in specific areas. Probing questions were asked to gain more information where necessary.

Interviews were audio recorded using a phone and laptop. Permission to record was sought from participants in the consent form and verbally at the beginning of the interview. The interviews were manually transcribed by Murray Transcription.

### Data analysis

Participants were characterised according to their involvement in the Partnership: Members of the *Strategic Partnership* (SP), implementation team (I) and/or research team (R); or food industry stakeholder engaged through the Partnership intervention (Fig. 1).

Transcripts were de-identified and imported into NVivo for data management. Transcripts were thematically analysed by one researcher (E.R.), using a combination of deductive and inductive methods, with input from the research team (W-K.C., B.M., K.T. and J.W.). The Consolidated Framework for Implementation Research (CFIR), was used to guide the qualitative analysis of the interviews to better understand factors affecting program implementation [17] within the context of the main process evaluation framework [12]. The CFIR is a comprehensive framework designed for evaluations of interventions to understand the effectiveness of intervention implementation and specifically “what factors influenced implementation and how implementation influenced performance of the intervention” [17]. The CFIR consists of five domains: Intervention characteristics (e.g. design and development of the intervention), outer setting (contextual factors affecting intervention design, implementation and outcomes e.g. political and social factors), inner setting (e.g. networks and communication within the Partnership), characteristics of individuals (e.g. knowledge and beliefs about the intervention) and the process of implementation (e.g. execution of the intervention). To enable a more in-depth analysis of the CFIR process domain, elements of the broader process evaluation framework were incorporated [11]. Constructs included in this analysis were: Stakeholder perceptions on achieving intervention aims (e.g. reach and dose), and fidelity and quality of the intervention. The CFIR domains and adapted constructs used in this analysis are displayed in Table 1. To ensure all meaningful data were captured, an inductive approach, line-by-line transcript analysis, was used to identify themes not already captured by the framework [18].

**Table 1:** Consolidated Framework for Implementation Research (CFIR) domains and adapted constructs

Domains	Adapted constructs^a^
Intervention characteristics	Intervention design and development
	Relative advantage
	Adaptability
Outer setting	Cosmopolitanism
	External policies and incentives: National level, state level
	**Other outer setting factors: Political context, socio-cultural factors, environmental factors, technological factors.**
Inner setting	Structural characteristics: **Partnership structure, organisational roles and responsibilities, changes in personnel, organisational changes**
	Networks and communications: **Collaboration, communication**
	Implementation: Compatibility, learning climate
	Readiness for implementation: Available resources
Characteristics of individuals	Knowledge and beliefs about the intervention
	Self-efficacy
	Individual identification with Partnership
Process	Planning
	Engaging the right stakeholders: Opinion leaders, formally appointed internal implementation leaders, champions, external change agents
	Executing the intervention: **Achieving goals, enablers and barriers to delivery, fidelity, utility/quality**
	Reflecting and evaluating

^a^Bold indicates additional construct

### Ethics and consent

This study was approved by the University of Sydney Human Ethics Research Committee (2016/770). Written informed consent was obtained from all participants prior to the interview.

## Results

### Sample

Twenty-four stakeholders from 11 partner organisations and 19 food industry stakeholders were invited to be interviewed. Sixteen Partnership stakeholders agreed to be interviewed, two declined as they felt they were not an active member of the partnership, one declined as they felt a colleague (already invited) was best placed to participate, two were no longer with their organisation, and three did not respond. An additional two stakeholders agreed to participate however did not complete the interview process. Seven food industry stakeholders agreed to be interviewed, two declined as they no longer worked for the company engaged by the Partnership, and ten did not respond.

In total, 14 Partnership stakeholders and seven food industry stakeholders were interviewed. Of the Partnership stakeholders interviewed, 12 were part of the *Strategic Partnership*, five were part of the implementation team and eight were part of the research team. Seven participants were members of more than one group and two were members of all three groups (Fig. 1). Of the seven food industry stakeholders, one was employed by a major retailer and 6 were from large food manufacturing companies [19]. The average duration for Partnership interviews was 39 min and for industry interviews was 30 min. In general, food industry stakeholders were only able to speak about the industry engagement arm and were not aware of the broader Partnership strategy.

**Fig. 2:**
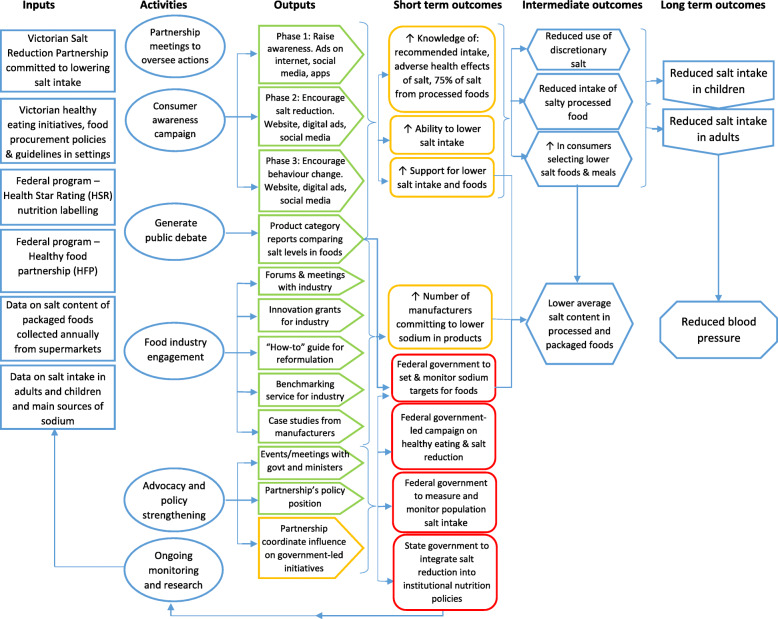
Revised logic model of the Partnership program. Adapted from Trieu et al. [12]. Green box – stakeholders thought this was achieved. Amber box – stakeholders thought this was partially achieved. Red box – stakeholders thought this was not achieved

In the subsequent sections, we describe the key themes from the stakeholder interviews using the CFIR adapted constructs with supportive quotes. Themes are organised as follows: (1) the Partnership (inner setting, intervention characteristics and individual characteristics), (2) execution of the four intervention arms including achieving intervention aims (e.g. reach and dose), enablers and barriers to execution, and fidelity and quality of the intervention (process), (3) contextual factors affecting intervention design, delivery and outcomes (outer setting). An overview of the Partnership program, and stakeholders’ perceptions on the effectiveness of the intervention, is illustrated in the revised project logic model (Fig. 2).

### The Partnership

#### Perceived effectiveness, structure and function of the Strategic Partnership

The establishment of the *Strategic Partnership* was described as one of the biggest successes of the project. Stakeholders believed that the *Strategic Partnership* was effective in achieving its goals of capacity building through the transfer of knowledge, skills and expertise between members, and collaborative action through the development and execution of a shared action plan. The *Strategic Partnership* was viewed by most as essential for intervention planning and decision-making processes that underpinned the execution of the intervention and perceived to be a “background enabler” for intervention delivery (Table 2).

**Table 2:** Stakeholder quotes illustrating key themes for each process element, by action area

Action area, aim and approach	Process element	Theme	Quotes
Establishing a Strategic Partnership Aim: to create a Strategic Partnership and develop a collaborative approach to salt reduction with stakeholders Approach: VicHealth invited stakeholder organisations to join the Partnership and develop a joint action plan for salt reduction in Victoria.	Perspectives on achieving aims	Learning how to work together	*It’s a true partnership where every organisation brings strengths to the table... the real success of this project is that, learning how to collaborate and work with others and recognise each of the strengths that they bring is really important. (Member 9: SP, R)*
		Achieving collaborative action	*I think the partnership had achieved what it set out to achieve in terms of collaborative action and setting of action plan that is about us working together for salt reduction specific to the state. (Member 11: SP, I, R)*
		Achieving capacity building	*I think there has been good ongoing discussion, collaboration, I think there’s been a bunch of capacity building. (Member 4: SP, R)*
	Enablers to intervention delivery	The “right” Partners	*I think you had the right organisations, strong leadership within those organisations and good expertise. (Member 2: SP, I, R)*
		Commitment from Partners	*I think that’s been critical in having all of those people with a common interest around the table because it’s been such a long time, the working group period, and you need a range of players committed to that to keep momentum going through. (Member 1: SP, R)*
		Regular strategic meetings	*The ability to have a meeting every three months, same people coming together, who heard the conversation last meeting, at that partnership level – it usually makes it much easier and it’s kind of an enabler. (Member 11: SP, I, R)* *The regular meetings were really good opportunities, they were well-run and focused and so I think they were fantastic opportunities to build momentum in the salt space and the hypertension space, we really hadn’t had something like that for a really long time, so that was terrific. (Member 8: SP)*
		Diverse skills and expertise (capacity building)	*I think those core groups brought a really good mix of skills and expertise, which I think we all learnt from. In my early days on the partnership when we’re establishing the agenda, it was really useful and great because we all learned from one another and you know we had some pretty fiery kind of debates and discussions, which were great. (Member 7: SP)*
		Developing a shared action plan	*Looking at the evidence, engaging with the key stakeholders around it, appraising options for action, feasibility, political acceptability a whole host of different domains to then draw-up a shared plan, on what the consensus for action on that would be that everyone could co-commit to. (Member 13: SP)*
	Barriers to intervention delivery	Less active involvement from some partners	*I think we’ve had some who haven’t actively contributed in the same way as others... they share our messages and our campaign, but it wouldn’t always feel like they had a stake. (Member 11: SP, I, R)*
		Too many people / organisations	*You do have to get a lot of people involved and a lot of people across things or signed off by people. (Member 5: SP, I)*
		Slow start-up	*It might have been a good year before it was quite clear what they were planning to do to me … trying to design implementation strategies… looking at getting other stakeholders involved and it is quite a slow process, but it took a while for me to clearly understand what the implementation strategies were going to be. (Member 3: R)*
		Transfer of knowledge and skills to new members	*We could have done more in terms of really making sure the new people who came in were fully aware of what the precise goals of the partnership, the ways of working and that we did everything we could to ensure that the capacity was transferred for the relevant people…I think sometimes you just underestimate the benefits or stopping, taking stock, making sure that absolutely everybody in the partnership is on the same page and has all the relevant background information and knowledge and skills in order to ensure that the project continues on the same track, and I think potentially we could have spent some more time doing that. (Member 2: SP, I, R)*
	Barriers to achieving outcomes	The “right” intervention	*I think the Heart Foundation to the extent that it delivered an intervention, did deliver an intervention pretty effectively, but whether the intervention was the intervention that needed delivering I think is a key question. (Member 4: SP, R)* *Everyone’s passionate who was around the table on this issue and of course you’re going to have some heat, and that’s part of it, it shouldn’t all just be smooth sailing, you need people to question, challenge whether you need the consumer piece or do you just focus on the food reformulation or do you do the debate and the policies? (Member 7: SP)*
	Perspectives on the fidelity of the intervention	Changes to Partnership members	*A number of other players have come on as it’s deemed relevant that they can contribute, and obviously they have an interest in this area. (Member 1: SP, R)*
	Perspectives on utility/ quality of the intervention	Effective Partnership	*It’s been really effective to have these different organisations with different skillsets working so closely together. (Member 21: SP, I)*
		Enabler to intervention delivery	*The Partnership’s a background enabler. (Member 12: SP)*
Raising Consumer Awareness Aim: to increase public awareness of salt intakes, salt and health, dietary sources of salt and steps to reduce salt intake. Approach: a three-phase digital campaign that targeted primary food providers within families with primary school-aged children.	Perspectives on achieving aims	Positive achievements, given limitations	*With the campaign, they’ve done well, given the budget and all the other factors, that was always going to make that a tough task. (Member 10: SP)*
		Good reach and recall within the target audience	*Nearly 50% of the target markets were reached with this campaign across the three years that we did an intervention, which is huge, and then of that, 82% of that are recalling one of our top key messages about that there’s processed food is high salt, that they need to be reading labels and looking for lower salt options, and eating fresh is best. So, there’s some really good positive outcomes and they were achievements against objectives. (Member 21: SP, I)*
		Limited impact on general population	*The metrics we’ve got on awareness-raising were promising and that’s great but Deakin’s research would say we’re not changing knowledge, attitudes and behaviours on a general public level but potentially with that target market of campaign we have raised awareness. (Member 11: SP, I, R)*
		Concerted campaign	*We were able to run a concerted campaign for four years around getting Victorians to reduce their salt intake - I think that was a huge achievement. (Member 9: SP, R)*
	Enablers to intervention delivery	Utilising earnt media to increase reach	*Whilst all our paid advertising focused on Victoria, all of our organic social and earnt media was all national, so we reached far beyond that. (Member 21: SP, I)*
	Barriers to intervention delivery	Limited mediums	*Look I think [the reach] is limited to some degree because it’s only social media… there’s no broader brush...we haven’t got a diversity of mediums. (Member 7: SP)*
		Weak messaging	*Our call to action in our consumer awareness has perhaps not been as strong as it could’ve been. (Member 7: SP)*
	Barriers to achieving outcomes	Inability to cut-through other nutrition messages	*I think the target audience that we were targeting was problematic in that they’re being bombarded by all sorts of messages around what to feed their kids and how to feed their kids and how to get them exercising or how to maintain their health. So, I think there were some challenges in perhaps the target audience that we selected. (Member 9: SP, R)*
		Target audience did not see the relevance	*I think we’ve had problems with the target audience for the consumer group and I don’t know whether they were the best place to start...You can often find salt and that issue with the older age group because they see it as relevant to them and we’ve seen that through the consumer awareness that the people that were the biggest uptake are those that are older because they see the relevance. (Member 7: SP)*
		Short intervention timeframe	*All credit to the partners and the Partnership in that we were able to do this for four years but shifting consumer behaviour and changing an entire food supply is a real long-term commitment and you’re not going to see significant shifts in the space of four years. (Member 9: SP, R)*
	Perspectives on the fidelity of the intervention	Decision to try and move the target audience along the Stages of Change	*At first, we sort of wanted to make them aware and say, “look there is a lot of salt in these packaged products, so we want you to be aware of that”. And then they were like “okay, maybe we are making them aware, we have done a few different campaign bursts, can we get them to consider this a bit more, consider doing something about this”. So then that was introduced, more of like potentially a swap message, and then by the time I came to it last year when I started, through discussions with the general working group and the Partnership group, it was sort of like, “look, why don’t we see if we can push people towards that behaviour change so can we actually get them to act, can we actually get them to do something about reducing their salt”. (Member 5: SP, I)*
		Reverted to awareness and consideration due to cost-efficiencies	*We decided to cut that action and acquisition part and just focus on awareness and consideration, because we knew with the budget we had, we could still retain and get good numbers and healthy traffic and cost-efficiencies in educating people at that stage (Member 5: SP, I)*
		Limited mediums after first campaign round	*The first round of the campaign, for “Unpack the Salt”, we did outdoor advertising, which performed well. We didn’t have quite as much money the second time we ran it, so it was then more just digital and online... When you change because of finances, you might lose a little bit of your impact. (Member 11: SP, I, R)*
	Perspectives on utility / quality of the intervention	Not effective in increasing public demand	*What we were hoping was that the campaign originally would generate some of the community awareness and action to help elevate [salt] as a conversation, a bit like it has with family violence and is starting to happen with obesity. That hasn’t really happened and so the politicians have been able to kind of ignore it for want of another. (Member 10: SP)*
		Not effective at population salt reduction	*With the small number of people that that impacted then that was a good response, but it was a small number of people so at a population level I would say it was ineffective (Member 10: SP)*
Generating Public Debate Aim: to generate public discourse and debate around salt reduction Approach: media releases disseminating product category reports that highlighted salt levels in processed foods throughout the intervention period.	Perspectives on achieving aims	Salt in the public domain	*I think we’ve had salt talked about in the public domain, pretty consistently, and I think that’s a really competitive environment for nutrition, so I think that’s a pretty great achievement. (Member 11: SP, I, R)*
		Shaping public debate	*I think the research has been particularly powerful and productive. It’s been a major part of the partnership and I think the quality of the research coming out, and the way in that’s being used to try and shape public debate, has been highly effective. (Member 13: SP)*
	Enablers to intervention delivery	Naming companies	*Those research reports, the way that they were designed to actually name companies and brands, meant we got great media pick-up, that meant we got great reach. (Member 11: SP, I, R)*
	Barriers to intervention delivery	Alignment of organisational and Partnership views	*The Heart Foundation is, as are most organisations are now, shifting away from the nutrients focus… to a more whole-food approach. So, we certainly yes felt some tensions there… when we’re looking at food categories (Member 21: SP, I)* *Some of the food categories that VicHealth and the George Institute would have liked to go out with didn’t sit well with the Heart Foundation’s philosophy, so [the Partnership] couldn’t pursue those opportunities. (Member 21: SP, I, R)*
	Barriers to achieving outcomes	Crowded nutrition space	*The newspapers are full of debates about ‘clean eating,’ and caffeine will give you this and your processed fats will give you that, we used to talk about fats and now we’re talking about sugars. In that context, talking about salt it was always going to struggle to get cut through. (Member 13: SP)*
	Perspectives on the fidelity of the intervention	Changes in the design of the intervention	*If we now went into designing this, we’d go in with the intention of implementation looking like it ended up... It took a while to get to that... this idea if you benchmark industry around a nutrient or around the healthiness of their products, you can leverage them for public awareness and food industry engagement and potentially government policy engagement depending on what the political landscape is. That’s a key lesson. (Member 11: SP, I, R)*
	Perspectives on utility/ quality of the intervention	Engaging media, industry and policy makers	*What’s been really effective has been the product category reports, which the George Institute has done. These regular reports, which basically look at salt levels of certain foods, we use those for strategic advocacy to get media attention... it raises awareness of the public and our policy makers, but it also enables us to liaise directly with the food companies to raise their awareness... and invite them to the table, and to develop strategies to try and reduce salt ... that’s worked relatively well. (Member 2: SP, I, R)*
		Scalability	*This concept of doing these surveys on food groups and resurveying every few years and that naming and shaming of individual products, brilliant, absolutely critical and I reckon that was something that would be scaled up and a really valuable piece of work. I thought that approach was very important. (Member 8: SP)*
Innovative approaches with food industry Aim: To reduce salt levels in packaged and processed foods by supporting food manufacturers to reformulate. Approach: Engage food industry through events and meetings, develop resources, services and grants, and produce case studies.	Perspectives on achieving aims	Establishing relationships	*We’ve been successful in developing really strong relationships with some of Australia’s major food manufacturers (Member 6: I)*
		Understanding reformulation practices	*We set out to engage with top manufacturers and understand what’s happening within these businesses and then to showcase their practice, where it was available and try and help those who aren’t, and I think we have definitely achieved all of those things (Member 21: SP, I)*
		Developing supportive resources and services	*And we also were able to, as a result of working with [the food industry], develop a guide to support particularly the Victorian-based objectives that we originally had at the outset of the project. So that guide was something we could develop for these smaller to medium manufacturers based in Victoria and that’s been shared with them through various networks. So yes, I think we’ve definitely achieved those outcomes. (Member 21: SP, I)*
		Changes to the food supply	*How effective will activity that has kind of sped up in the last year or two be in actually getting salt levels down in the time that we’re measuring?... If you looked at it right now, I’m not sure if we could see that the food supply has already shifted. (Member 1: SP, R)*
	Enablers to intervention delivery	Building positive relationships	*Your overall aim was to really support manufacturers and you wanted to provide us with the reformulation guide, you had an event that was really, really supportive of companies who were doing things well and that really helped us to get involved. (Industry 15)*
	Barriers to intervention delivery	Uncertainty of roles and authority	*This is happening at Federal level and therefore what is our role and what is our authority in terms of industry engagement. So, I think it was that lack of clarity of roles, and where do we fit and what’s our authority, I think that was probably one of the barriers. (Member 2: SP, I, R)*
		Slow start to implementation	*Engaging with food industry at the start may have been a bit slow but I personally don’t have experience engaging with food industry. Other people at the table have some, but I think it’s taken a while to probably come up with the working plan of what is the best approach to engage with that target group. (Member 1: SP, R)*
	Perspectives on the fidelity of the intervention	Changes to the plans due to new knowledge and innovation	*Overall the Partnership has been so open to trial and error and the level of innovation in terms of let’s try this and let’s try that. The resources that have been developed by the partnership for industry, we never envisaged that at the beginning. Who would have envisaged two years ago that we would have finished this project with a how to guide, benchmarking reports and grants? (Member 6: I)*
		Changes to the target audience to effect more change	*We changed half-way through in regards to our thinking around who we should target.... If we had been thinking at the start around how can we effect the most change and target the big ones with these food category reports and then how do we effect the most change in Victoria by providing tools and resources and contacts for small to medium, that would have been a nice round brand from the start. (Member 21: SP, I)*
	Perspectives on utility/ quality of the intervention	‘Reformulation Readiness’ Guide	*The reformulation readiness guide... I think that is a great resource that was put out... We had a lot of good feedback from different areas of our supply chain and food technologists around that guide, so just really helpful. (Industry 17)*
		Industry-centred events	*It’s probably the first time we have seen a public health organisation run an industry event and be supportive... you understand the work we are doing, and you’re willing to support it and highlight where companies are doing well (Industry 15)*
		Case studies	*Smaller businesses... they need to see what we’re doing and they need to learn from us and if they see case studies from us, that can really help them shape their nutrition strategy, so I think it’s a positive for those other industry players and also consumers to see what we’re doing and obviously government to see what we’re doing (Industry 17)*
		Benchmarking service	*The George Institute was offering those reports which could take one companies’ products and compare it to the entire category... I think that’s really valuable as well because you know, all companies are going to be interested to know what their competitors are doing. (Industry 16)*
		Reformulation grants	*The salt reduction grants, I think that’s showing really strong leadership and showing food manufacturers that there is support and there are resources available to do this. (Member 6: I)*
		One-to-one meetings	*We’ve been able to undertake over 20 one on one engagement meetings which have revealed a lot of insight and information around the drivers for reformulation and the current obstacles and issues that food manufacturers are still facing. (Member 6: I)*
Advocacy and policy strengthening Aim: to “strengthen healthy policies” to improve the food environment, including food reformulation programs and institutional nutrition policies. Approach: Engaging government stakeholders, creating a policy position statement and influencing government initiatives.	Perspectives on achieving aims	Did not achieve policy change	*I reckon if we’d just gone, “You know what, to make an impact in Victoria what we need to do is we need to change things nationally,” we just spent all of our time and effort just focusing on bringing the Victorian State government, regulators, power and influence to bear on Canberra and indeed on the other states, to actually change things for the whole country, maybe that would have been more successful. (Member 4: SP, R)* *Whilst we might not have seen policy change, we’ve definitely continued the conversation and put support behind it. (Member 21: SP, I)*
		Supported already planned activities	*I feel like the advocacy activities probably have just added a strong supporter base around things that were in-train. (Member 11: SP, I, R)*
	Enablers to intervention delivery	Developing a blueprint	*We developed the call to action document. I think was a great output and a deliverable... it’s probably the first-time organisations have got together to actually get some form of consensus, a blueprint on what we need going forward. (Member 7: SP)*
	Barriers to intervention delivery	Lack of allocation of roles and responsibilities	*We developed a clear statement, which outlines what the key asks of the strategic partnership were, but then we didn’t identify peer roles or allocate specific responsibilities to the different partnership members in terms of who was going to take that forward. (Member 2: SP, I, R)*
		Uncertainty around ownership and leadership	*I think one of the challenges of the Partnership, when you’ve got multiple organisations, a lot of strengths, but then you’ve got a certain degree of who’s owning it, leading, driving it, and who’s going to make those calls and who’s going to get the credit for it. Which is unfortunately the politics working anywhere and I think that’s probably held some of the progress back in that policy advocacy space because mixed in with all that political context. (Member 10: SP)*
	Barriers to achieving outcomes	Political climate	*There’s been some good work but as a whole it probably hasn’t quite had the impact at the state and federal level as we would have wanted. That’s not necessarily because of the fault of any of the partners, it’s partly because of the political conversations and agendas out where salt is and you can’t make an issue popular with politicians if they don’t want it to be and there’s not a public push. (Member 10: SP)*
	Perspectives on the fidelity of the intervention	Shift from targeting state government to federal government	*Up until probably June 2018, we were advocating at a state level, and then we shifted, we thought it was more of a national, a federal issue... so we wrote a new plan (Member 21: SP, I)* *At the very beginning of the project, it was very Victoria focused… but along the way the decision was made… it’s a national thing, if they’re going to make that policy, it’s going to be Federal policy. (Member 5: SP, I, R)*
	Perspectives on utility / quality of the intervention	Joint advocacy	*There are lots of benefits to a partnership working together on strategic advocacy and I think we’ve utilised that quite well on some occasions, but perhaps not as well as we could throughout the whole project, and I think this is probably a challenge for these sorts of partnerships in general. (Member 2: SP, I, R)*
		Advocacy asks document	*Some people might say well that’s the statement of the obvious but to have it as a document there, to hand-out to governments and to use as and advocacy tool was a milestone. (Member 8: SP)*

Interviewees felt that having the “right” organisations and individuals involved, with a diverse range of skills and expertise, and creating a positive learning climate, where they were able to share ideas and provide input into the strategy, were important components for designing and delivering a feasible, evidence-based salt reduction intervention in Victoria*.* Strong engagement from organisational leaders and *salt champions* were enablers for establishing and sustaining momentum and enthusiasm for the project. The commitment of members of the *Strategic Partnership*, demonstrated through their consistent attendance at quarterly strategic meetings, was viewed as an enabler to achieving strong communication, joint decision making and collaborative action (Table 2).

Most members spoke positively about the experience of being involved in the *Strategic Partnership* and the development of the shared action plan. Though one stakeholder shared that there was some healthy dispute between members. A few stakeholders questioned whether the agreed strategy contained the right interventions. They suggested that the intervention arms aiming to change the food environment, such as *Industry Engagement*, should have received greater focus and resources (Table 2).

Differences in perspectives on the Partnership structure and function illustrated important communication and knowledge gaps. Some of the implementation team viewed the role of *Strategic Partnership* as less important, likely because day-to-day functioning and decision-making was carried out at the level of the implementation team. A few interviewees proposed this communication gap was the result of a high turnover of staff within the implementation team and suggested more could have been done to transfer knowledge and capacity to new implementation team members through better on-boarding processes (Table 2).

These gaps resulted in a misunderstanding of the *Strategic Partnership’s* roles and responsibilities by some, which was illustrated in how interviewees described *Strategic Partnership* members contributions to the coordinated efforts for delivering the intervention. Interviewees perspectives on the roles and responsibilities of key partner organisations are displayed in Table 3.

**Table 3:** Organisational roles and responsibilities, as described by interviewees

Organisation	Roles and responsibilities
VicHealth	*VicHealth has clearly set direction and part of the reason we’ve had that role is because we have funded the implementation. (Member 11: SP, I, R)*
Heart Foundation	*Heart Foundation was awarded a contract by VicHealth to deliver three areas of the intervention, being consumer awareness, advocacy and industry engagement (Member 21: SP, I)*
The George Institute	*The George Institute was engaged for strategic advice and also as a research partner and subsequently built an NHMRC partnership grant around the work which the partners were engaged on (Member 13: SP)* *As we move forward, we have been supporting the Heart Foundation in terms of the intervention’s strategy. (Member 2: SP, I, R)*
IPAN at Deakin University	*Much of our work has been collecting that baseline and end data for surveys in regards to knowledge, attitudes and behaviours have shifted on salt consumption throughout after this intervention, as well as actual salt intake in children and adults. (Member 1: SP, R)*
Department of Health and Human Services Victoria	*Providing strategic advice and promoting the project broadly… contributing funding… government rep. (Member 12: SP)*
Other organisations on the Strategic Partnership	*Attend four meetings a year, we’re just looking for their input, looking for their contribution, looking for where they might be able to amplify communication messages (Member 11: SP, I, R)* *All of the aspects of the partnership plan were developed through consultation with myself and the other strategic leaders. (Member 13: SP)*

### Execution of the four intervention arms

The intervention action areas, process domains, key themes and stakeholder quotes relating to the execution of the intervention are displayed in Table 2.

#### Consumer awareness campaign

Stakeholder perceptions on the effectiveness of the *Consumer Awareness* campaign were mixed. Stakeholders involved in, or overseeing, intervention delivery shared that a digital campaign was strategically chosen for its cost effectiveness and efficiency in reaching the target audience. Eight interviewees expressed that the reach was “adequate” given the constraints of the chosen approach, including the budget, timeframe and target audience. Some of the intervention team shared specific achievements of the campaign, such as high recall of the key messages within the target population (Table 2).

However, overall, many felt the campaign was limited and did not have enough reach. Two participants stated that the campaign was ineffective in making a meaningful contribution to the overall Partnership goal of a one-gram reduction in population salt intake. Amongst those who expressed scepticism towards the campaign’s effectiveness, challenges in delivering this intervention arm and engaging the targeted population were discussed. Five reasons were suggested by interviewees: (1) The target audience did not see the relevance for them because of the perception that high blood pressure was an older person’s problem; (2) the target audience was “bombarded” with digital health messaging from other sources; (3) absence of hard-hitting campaign messaging that did not “cut-through” other digital health messages that flooded media and social media spaces; and (4) limited campaign mediums (digital only) as a result of tight budgets; and (5) the intervention timeframe was too short (Table 2).

#### Generate public debate

The primary mechanism used to generate public debate was perceived by interviewees to be an effective central lever for consumer awareness raising, engaging the food industry and pursuing advocacy and policy asks through media advocacy strategies. One stakeholder shared how the strategy was developed overtime and many interviewees didn’t differentiate it from the Consumer Awareness arm. Ten Partnership members discussed the utility of the product category reports in engaging media and industry. The reports were perceived to be a “star performer”, achieving more media and industry engagement than anticipated, and at low cost (Table 2).

However, the majority of interviewees (10 Partnership stakeholders and three food industry stakeholders) also acknowledged a competitive media environment, which was “full of debates about ‘clean eating’” and “sugar”, as something that potentially hindered impact. Implementation team members identified that some individuals and organisations had changed focus from salt as a single nutrient towards general healthy eating principles, as a challenge that had created a misalignment between individual, organisational and Partnership objectives and values resulting in friction between some members. These “tensions” were resolved; however, delivery of this intervention arm was slowed and perceived as “challenging” at times (Table 2).

#### Food industry engagement

The *Industry Engagement* strategy was viewed as effective in terms of innovative approaches to industry engagement and creating positive relationships between public health organisations and the food industry. However, both strategic partnership members and program implementers identified challenges, including perceived changes to the strategy during the intervention (Table 2). Stakeholders cited a slow start to implementation of this intervention arm. A few suggested this was due to an initial lack of understanding of the food industry, how to engage them and which organisation would be responsible. Some interviewees said that the focus initially was on supporting small-to-medium manufacturers in Victoria to reformulate processed foods, but this was later expanded to include national and international manufacturers, in order to have an impact on the Australian food supply (Table 2).

Stakeholders spoke positively about the learning experience and the process of adapting the strategy to address industry needs identified throughout the intervention. Both Partnership members and food industry stakeholders spoke of the strong positive relationships that were created. One-to-one meetings were seen as important for building rapport and a two-way exchange of knowledge between the Partnership and food industry. Interviewees discussed how through these meetings, new knowledge and understanding of industry levers and capabilities were gained, which enabled the Partnership to adapt the industry engagement strategy to utilise levers and create services and resources to fill identified gaps in capabilities. This included: (1) the development of a guide for reformulation to support companies to undertake reformulation, (2) a benchmarking service to assess the nutritional profile of products and compare to competitors and to sodium targets, and (3) grants for small-to-medium manufacturers to financially support reformulation. In addition to increasing capacity, interviewees shared that the Partnership was able to demonstrate supportiveness of companies’ positive progress towards sodium reduction through public case studies and showcases at events. This was perceived to further facilitate relationship building and help overcome the “us and them mentality” between public health organisations and the food industry (Table 2).

#### Advocacy and policy strengthening

Stakeholder perspectives on the effectiveness of the *Advocacy and Policy Strengthening* arm were mixed, likely due to differences in understanding of the intended objectives and strategy. Two program implementers shared how the policy and advocacy activities were initially carried out at a state-level, however the Partnership identified that key levers for certain food policies were at different levels of government and that there was a mis-match between what was planned and what was achievable at a state-level. Specifically, stakeholders spoke about food reformulation programs being a federal matter and many suggested this advocacy could have been more successful if it had a national focus from the outset, though some recognised the challenges associated with the funding coming from the state for a Victorian-specific intervention (Table 2). Contrastingly, one strategic member spoke of the planned strategy as always intending to target both the federal and state governments and two strategic members shared that very little progress was made towards integrating salt reduction into state-based policies, including institutional nutrition policies.

Participants talked about the development of an advocacy asks document, that was perceived to be vital in bringing the organisations together and forging a consensus on the way forward for the Partnership. However, the utility of the document, and indeed the overall strategy, was a contested topic, with some viewing it as a “milestone” and others a “statement of the obvious”. Many shared how the Partnership had “affected things that have been going on”, such as providing feedback on the draft sodium reduction targets proposed by the Australian federal government’s Healthy Food Partnership in 2018 (Table 4). However, some stated the main advocacy ask of getting the government to establish targets for salt was not achieved within the intervention period. Many identified a lack of coordinated advocacy actions and clear allocation of roles and responsibilities to have hindered intervention delivery. Uncertainty regarding which organisation was driving the strategy and when organisations were advocating as part of the Partnership or alone were suggested to have held up intervention delivery and led to sparse communication of advocacy activities between members, resulting in a lack of coordinated action. The lack of allocation of roles created uncertainty amongst individual members as to who was responsible for carrying out the advocacy activities and who was responsible for making sure they were executed (Table 2).

**Table 4:** Contextual factors affecting intervention design, delivery and outcomes

Contextual factors	Theme	Quotes
Policy environment	Healthy Food Partnership’s slow progress towards setting draft sodium targets	*I feel like the advocacy activities probably have just added a strong supporter base around things that were in-train. A reformulation programme was being discussed, being designed, being consulted on. We could then put Partnership responses in, the technical response that The George Institute put in, or just put the weight behind it – we need it fast, we need it to be a really comprehensive programme. (Member 11: SP, I, R)*
		*I think we’ve certainly affected things that have been going on. The Healthy Food Partnership was there, and we were able to offer commentary whenever we were asked, we offered support and we will continue... to kind of push that reformulation story with government. So, whilst we might not have seen policy change, we’ve definitely continued the conversation and put support behind it, extra support behind it when we’ve been asked. (Member 21: SP, I)*
		*It was challenging to get engagement because the food companies were waiting to find out what the salt reduction targets were going to be… the Heart Foundation, with our support, manages to identify a program of work to engage industry at the same time as waiting for the targets. (Member 2: SP, I, R)*
		*Obviously, the lag on the targets for the Healthy Food Partnership being set has been a bit disappointing and obviously that’s outside the control of the group. Once those are in place you would think that that may heighten the activity of the food industry and manufacturers to reformulate and help to reach that outcome but again that’s still a while away. (Member 1: SP, R)*
	Alignment with state nutrition policies/ strategies (e.g. Healthy Choices)	*I don’t think [salt] is necessarily embedded in strategy at [state] government level... there’s a few individuals that have really driven it and championed it which has been fantastic, but whether that’s embedded in the strategy if they were to leave, I’m still not sure. (Member 7: SP)*
		*In relation to the state-wide policies on food in institutional settings, it’s been a challenge to impact on those processes and also to know whether or not those policies are being implemented and whether or not they are also incorporating salt in those policies...we didn’t have any direct access to those institutional settings. (Member 2: SP, I, R)*
Political climate	Salt is not a current government priority	*There’s been some good work but as a whole probably hasn’t quite had the impact at the state and federal level as we would have wanted, and that’s not necessarily because of the fault of any of the partners, it’s partly because of the political conversations and agendas out where salt is and you can’t make an issue popular with politicians if they don’t want it to be and there’s not a public push... so the politicians have been able to kind of ignore it. (Member 10: SP)*
		*We obviously wanted policy change. We have had a government in ... it’s not the highest thing on their priority list. (Member 21: SP, I)*
	Lack of policy window in the intervention time period	*It would have to do with the fact that what we’re trying to do was trying to move a mountain and that’s a very difficult thing to do when you’re just a group of mountaineers... The reality is it’s still a priority, it needs to be a priority and like in tobacco control or any other public health area, often it can take twenty or thirty years for the advocacy really to reach a critical mass and then find a sympathetic minister or a sympathetic government or a sympathetic community and the timing is right and suddenly you get an opportunity… A lot of success of public health has time to build and a long time to make the business case, and then the policy window opens, and that’s the opportunity there. (Member 13: SP)*
		*Sometimes you’ve just got to go with what the political climate’s going to let you do, and both with industry and politicians and sometimes it’s not your day or year or your decade and you can’t change everything unfortunately. (Member 10: SP)*
Social factors	Crowded nutrition space	*The reality is that the broad community debate on these issues have usually been dominated by issues of a sugar tax or health levy on sugary drinks and the role of sugar as a driver of the obesity epidemic in recent years, and I think to that extent the Partnership and the salt focus was always going to struggle to get cut through, but it doesn’t mean that it’s a failure, it just means that it’s a competitive environment for community engagement on food and I think there’s been some good progress made by the Partnership in general community awareness, engaging with the industry, engagement with policy makers as well but that’s against the backdrop of broadly a lack of recognition of salt as a population health priority in the Australian context. (Member 13: SP)*
		*Sugar was much more of a top of mind issue for many, particularly consumers, but also for industry and even government to some degree, or the political end of the spectrum. (Member 7: SP)*
	Perceived value of a single nutrient approach	*Not everyone in Victoria, and in Victoria healthy eating sector, would support a salt reduction approach... nutrient-specific projects are not really what they’re at the moment, prioritising. (Member 11: SP, I, R)*
		*I’ve always been a very strong internal advocate in the food and nutrition space and particularly around the need for a focus on salt reduction and it has been one issue that really was becoming a little problematic was this broader issue that’s run by nutritionists, in general, dietitians I should say, about the fact that we shouldn’t focus on single nutrients. (Member 8: SP)*
Environmental factors	The broader food environment	*Everyone in Australia gets 70% of their salt or 80% of their salt from packaged foods and restaurant foods. (Member 4: SP, R)*
Technological factors	Technology as a barrier to reformulation	*Barriers are taste, technology is to do it... consumer acceptance, quality because salt provides a technical role, not just boosting flavours, though that’s definitely a factor. (Industry 17)*
		*There are things that we can do and there are things that we can’t do... also the implications of reformulation, what do we replace it with, if anything, and is that more or less harmful than the original. From our perspective if we ask the food industry to reformulate in a short period of time, they can only use the technology that’s available to them, but you know doesn’t give them time to look for other options and you may get a food supply flooded with things that you don’t want. And that’s what we are very mindful of and we’re very concerned... we try not to put other things in that may not be necessarily a better option. (Industry 18)*
		*We also have joint research programs... to try and use different technologies so we can get the sodium down but still have a good taste. (Industry 19)*
Other factors affecting the food industry’s ability to reformulate	Functional role of salt in food	*In many cases, especially in extrusion and in other types of crisping, there’s only so much you can take out before the product no longer resembles its form. (Industry 18)*
		*In some other industries... you need salt for a functional benefit, and also in some of the products we do like crackers where sodium bicarbonate is used as a raising agent, it’s sometimes very hard then to get products low in acid sodium. Sodium can be used either alone or in combination with other things like sodium bicarbonate, like a functional base, like a raising agent. (Industry 19)*
		*There is a sweet spot where manufacturers can take out small amounts of sodium without too negatively affecting taste and function, and I think responsible food producers are working towards achieving that. In order to go further, which is really becoming creative with ingredients and flavours and so on to reduce the sodium, but still maintain terrifically tasty products for consumers... If they don’t have the food science product development teams to work with ingredients and flavours and so on to produce outstanding products with taste, with low sodium... what they will need is possibly assistance or direction with, and exposure to a skillset and information that will allow them to explore product development and reformulation at low sodium target levels. (Industry 14)*
	Feasibility of removing salt from food	*In the company’s that I have worked for or consulted to, they have literally just removed the sodium because their products aren’t very complicated, they haven’t had to look for alternatives. (Industry 16)*
		*The thing with salt is that it’s easier to remove and replace salt than it is to remove and replace sugar. So, it is something that you can do by stealth and train the palate slowly to get used to. And because there is very little bulk in salt it’s just easier to take out than some of the other ingredients. (Industry 18)*
	Skills and expertise to reformulate	*Understanding that some, especially the smaller manufacturers, might need some skilled food science and product development help in reformulating lower sodium, but still maintaining amazing mouth feel and taste. (Industry 14)*
		*Companies now employing dietitians and registered nutritionists. That’s important that they acknowledge that they need that level of expertise and science within their company to help guide them, to help guide their leadership team to what they should be doing from a health perspective. (Industry 17)*
		*The big players are the ones who are doing a lot in that space because they’ve got the resource, like we’ve got a dedicated nutrition team, a dedicated regulatory team, a sustainability nutrition team, we’ve got the resources. Whereas I know the smaller businesses don’t and they need to see what we’re doing, and they need to learn from us. (Industry 17)*
		*There’s a huge gap in resources and huge gaps in capabilities. Even the large manufacturers who we naively thought had the capabilities do not have well-resourced nutrition units within their organisations. (Member 6: I)*
	Cost of reformulating	*I think cost is a massive issue. Buying salt replacements is more expensive than putting salt in and it’s just how willing people are to be able to take a hit on growth margins, and if you do it slowly by stealth then you’re in a better position. (Industry 18)*
		*Every single reformulation that you do requires a project, requires resources, requires trial time, requires trial managing, requires package changes, it’s a very expensive exercise. (Industry 18)*
		*Salt is a cheap ingredient and it can be that as we reduce salt, the costs go up. (Industry 2)*

### Contextual factors affecting intervention design, delivery and outcomes

Stakeholders identified legal, political, social, environmental, technological and economic factors affecting intervention design, delivery and outcomes. Key themes and supporting quotes are provided in Table 4.

The state and national food policy environments were frequently discussed and perceived to shape the intervention design, hinder intervention delivery and prevent the Partnership achieving outcomes. At the state level, attempts to embed salt as a priority in current institutional nutrition policies (e.g. schools, hospitals and public sector workplaces) were thought by one stakeholder to be hindered by an inability to access relevant organisations to understand what programs were being implemented and a lack of available data on implementation or effectiveness. At the national level, slow progress by the government’s Healthy Food Partnership in setting and implementing nutrient reformulation targets was viewed as a barrier to effective action in two ways. Firstly, the announcement of the Healthy Food Partnership and its proposed program of work in 2016, required the Partnership to adapt the *Advocacy and Policy* strategy to the shift in the policy environment. Subsequently, the slow progress in setting targets, including a lengthy public consultation, impeded further advocacy attempts for policy change within the intervention timeframe. Secondly, the slow progress in target setting hindered *Industry Engagement* by the Partnership. Participants indicated that if targets had been in place there would have been an acceleration of industry reformulation activity and higher demand for the Partnership to support companies to reduce salt in food, and ultimately a greater impact on the food supply. Although one participant stated that the Partnership was able to put in place a strategy and “identify a program of work to engage industry at the same time as waiting for the targets” (Member 2) (Table 4).

The political and social climate was viewed as not conducive to achieving Partnership aims. Many stakeholders spoke about “a lack of recognition of salt as a population health priority in the Australian context*”* (Member 13). Some speculated that a crowded and competitive nutrition space in the public domain hindered the Partnership from achieving political and social traction, with sugar and obesity being more “top of mind” issues. Some spoke of the link between consumer demand and government and industry action. They suggested that a lack of public push for policy change continues to allow politicians to ignore salt as a health priority and a lack of public demand for healthy foods continues to allow industry to produce processed foods high in salt. A few stakeholders viewed a key role of the Partnership to be continued advocacy on the importance of salt reduction to consumers, government and industry, building a business case for when policy window opens (Table 4).

## Discussion

This research, based on in-depth interviews with 14 Partnership members and seven food industry stakeholders, has provided valuable insights into stakeholder perceptions on a partnership approach to implementing a salt reduction intervention. Use of CFIR enabled understanding of factors affecting program implementation and how these factors were perceived to influence Partnership effectiveness. This has added to our knowledge on successes and challenges of working as a partnership to deliver a multi-faceted public health intervention and provided valuable lessons for future initiatives.

### The Partnership

Stakeholders viewed the establishment of the Partnership as essential for intervention planning, design and development, and an enabler for intervention delivery. Previous nutrition coalitions in Australia have tended to be homogenous in knowledge and skills with most members being from a nutrition background [20]; whereas, effective health partnerships engage diverse members who bring a wide range of skill sets and knowledge [21, 22]. Consistent with this perspective, Partnership members viewed their diversity as a strength. The Partnership brought together skills and expertise in diverse areas such as communications, campaign management, public health interventions, disease prevention and treatment, advocacy and research. By working together, an effective and diverse partnership can achieve better outcomes than any individual or organisation can alone by sharing knowledge and skills, more-efficiently utilising available resources and executing joint activities [23]. Interviewees described how the diversity within the group facilitated the cultivation of a positive learning climate and they spoke positively about their ability to contribute to the strategy and their role in executing the Partnership’s plan. Thus, the effectiveness of intervention implementation was facilitated by the establishment of a Partnership with diverse members who were able to contribute their knowledge, skills and expertise, which enabled effective collaborative action and intervention execution.

### Execution of the intervention

The interviewees felt the planned activities of each intervention arm were executed and the intended outputs were generated, though some recalled adaptations and additions over the implementation period, and this was viewed as an achievement. However, there were varied beliefs regarding the translation of outputs to anticipated outcomes across the intervention arms [12]. This may reflect the delay between outputs and outcomes commonly seen in public health and policy interventions, but is also likely due to (1) internal factors, such as Partnership communication networks, (2) contextual factors, such as the social and political context, and (3) intervention fidelity, which were highlighted in the interviews.

#### Communication networks within the Partnership

The Partnership demonstrated some common features of effective collaborative frameworks, such as member commitment, communication structures, openness, planning, a shared vision, and clear decision-making processes [24]. For example, interviewees described clear benefits associated with being a member of the Partnership, such as fulfilling organisational health promotion goals, which were important in facilitating commitment to the Partnership and collaborative working [23, 25]. Stakeholders also described some qualities that could have been improved. Interviewees discussed communication challenges encountered between members working at the strategic level and program implementers. Despite quarterly strategic meetings, there was a lack of lack of clarity around the overall vision of the intervention, with stakeholders from these distinct groups holding different views of action area aims and objectives, and individual’s roles and responsibilities [22]. Hunter and Perkins propose that a lack of connection between members at the strategic-level and implementation-level is a barrier to effective partnership working due to a lack of transfer of knowledge, skills and expertise between groups [26]. They found that strategic partners were focused on achieving the strategic goals and targets, while those involved in intervention delivery operated in a more organic way, distinct from the objectives set at the strategic level [26]. This was evident within the current Partnership. Strategic-level members mostly spoke about achieving strategic aims and objectives that were determined at the outset, some of which were viewed as too ambitious; while program implementers seemed to have varied understandings of the strategic goals and were more focused on meeting organisational and individual contractual obligations (e.g. key performance indicators). The disparity between individuals’ perspectives on Partnership goals highlights a crucial gap between the expectations of the strategic partners and the ability of the program implementers to respond.

Furthermore, program implementers and strategic partnership members held different perspectives on each other’s roles. For example, strategic leaders viewed their role as central to the intervention design and delivery, while program implementers thought of the role of strategic partners as less important. Program implementers shared details about their decision-making processes, which tended to be made at the implementation team level and independently of the strategic goals. These decisions, combined with staff turnover and poor handover within the implementation team, resulted in subsequent changes to the intervention plan and ultimately changed the ways in which the intervention team thought about and executed the overall Partnership plan. Given shared understanding of clear aims and objectives, and delegation of roles and responsibilities, are factors that facilitate Partnership functioning [25, 26], the differences in individual perspectives that were highlighted in the interviews, may have reduced the effectiveness of the Partnership. Without having a solid understanding of the anticipated outcomes, the intervention team were working towards generating the outputs they were contracted to produce rather than striving to achieve the planned strategic outcomes through the outputs [26, 27]. This highlights the need for partnerships to ensure relationships and the transfer of knowledge and skills are maintained between those at strategic levels and those responsible for intervention delivery [26].

#### Contextual factors

The importance of planning and implementing an appropriate intervention for the context was highlighted, as well as planning within the constraints of a chosen intervention approach, such as within the limitations of a voluntary state-based program. This salt reduction project utilised a partnership approach to execute a four-armed salt reduction intervention plus monitoring and evaluation. Around the world, countries are employing multi-dimensional salt reduction strategies to reduce excessive population-level salt intakes and the associated disease burden [28]. The Partnership approach comprised evidence-based salt reduction strategies that have been previously identified, including: Measuring and monitoring salt use and knowledge, attitudes and behaviours of Victorians and salt levels in the Australian food supply, engaging the food industry to reformulate products, influencing the establishment of sodium targets for foods and front-of-pack labelling in Australia, consumer education to empower individuals to change their salt use behaviours and supporting the creation of healthy food environments in public institutions [29]. Simply implementing a multi-faceted evidence-based intervention is not enough to achieve public health outcomes in a complex real-world setting where contextual factors are at play [27]. Tseng suggests it is not just about what works, but “what works for whom, under what conditions and at what cost” [30]. Two key challenges were identified: executing a salt reduction strategy within one Australian state from a state-based perspective and lack of available policy levers stemming from the delegation of power between state/territory governments and the Commonwealth government in Australia. Legislative and regulatory responsibilities for health policy and health care are split between state and Federal governments [31], which has resulted in challenges executing public health responses both now and in the past [32]. The delivery of the *Policy and Advocacy* arm was hindered by complexities around this division of power, where some desired activities needed to be executed at the federal-level (e.g. advocating for sodium targets) and others at the state-level (e.g. integrating salt reduction into institutional nutrition policies). Partnership resources for this intervention arm were divided and stretched between the two approaches, and ultimately this resulted in a greater focus on Federal government advocacy and inattention to the state-based goal. Notably, at the Federal level, the few government strategies that have aimed to improve population diets have been voluntary and are unlikely to have much impact [33, 34], even though systematic reviews and modelling studies suggest mandatory or legislative approaches may be more effective [35, 36]. Regardless of this, through its program, the Partnership was able to engage with the food industry, including successfully building positive relationships and facilitating reformulation [7], in the absence of regulation. This example demonstrates that the Partnership approach and strategy could be a useful model for the Government to adopt. A key lesson from the Partnership was the need to understand the context of the intervention – the legal and political mechanisms that influence public health agendas – in order to plan an appropriate intervention that fits within the context and engages with these mechanisms, prior to commencing the intervention.

Further to this, the political and social climates were viewed as barriers to achieving planned outcomes; particularly policy change, given the lack of public and political push for salt reduction. To achieve nutrition policy change, gaining public support, and demonstrating it to policymakers, is crucial for generating political will [37]. As a newly-formed coalition, the Partnership firstly needed to gain credibility and trust from the public [37]. This is often hard for nutrition coalitions to achieve as there is a perception from the public that the nutrition message is constantly changing and there is lack of consensus among nutrition professionals [37]. Members believed a focus on salt reduction alone made it difficult to compete with more topical issues such as sugar and obesity. The public nutrition space was described as “crowded” by mixed messaging from various special interest groups making it difficult for the intervention’s messages to stand out and for the Partnership to build a credible reputation, which likely limited its influence on public will. Interestingly, even with substantial support and longer-term advocacy efforts, public health and nutrition professionals have not managed to influence the government to implement a national nutrition policy [38]. It is important to note, that the Partnership was able to influence the government’s sodium target setting process, and subsequent to the intervention (May 2020), the government announced 27 sodium reduction targets [39]. This suggests public health and nutrition coalitions can influence policies already on policymakers’ agendas but illustrates the challenge in generating the public and political will needed to get policies onto decision-makers’ agendas.

### Fidelity of the intervention

A monitoring and evaluation process was actualised to ensure intervention fidelity and generate the evidence needed to support decision-making to optimise the effectiveness of the Partnership intervention throughout the implementation period. Our analysis revealed that the program implementers were able to incorporate feedback and adapt components of the intervention plan in response to Partnership monitoring and evaluation efforts [14]. For example, the need for a more strategic approach that would have greater impact on the food supply by targeting both small and medium businesses as well as larger manufacturers through a variety of different engagement methods was identified in an interim assessment [14]. Effective partnerships have clear strategic plans, which are supported by robust monitoring and evaluation procedures, to determine how the partnership is performing and how intervention delivery is going throughout the intervention to establish if any modifications are needed [26]. Planned and unplanned adaptations are often made prior to interventions (e.g. for a specific audience or context) and during interventions (e.g. to optimise effectiveness) [40]. Adaptations to intervention plans can have a positive or negative impact on the intervention’s effectiveness [41, 42]. Following the intervention, stakeholders viewed the food industry strategy adaptation as one of the key successes and a facilitator in achieving Partnership outcomes. However, not all recommended changes were executed by the implementation team, which suggests there were missed opportunities to increase the impact of the Partnership intervention. For instance, in 2017 and 2019 stakeholders identified a need for stronger, strategic consumer-messaging that conveyed the serious health risks of high salt [14]; however, perceptions on the strength of awareness-raising messages remained unchanged throughout the interventions. Program implementers did not comment on why such feedback was not incorporated; however, this was potentially due to a change in key personnel. They did speak about an unsuccessful attempt to shift consumers along the Transtheoretical Model stages of change [43] through the three campaign waves. While the Transtheoretical Model can be applied to public health interventions to try to accelerate the rate of behaviour change, only a small proportion of the population will be willing to contemplate making a change and an even smaller proportion will be ready to take action within the intervention timeframe, leading to varied successes in changing dietary behaviour in populations [44]. This is an example of prioritising intervention fidelity over adaptation and optimisation, and raises questions about whether the Partnership could have made further progress towards its consumer awareness goals if the interim feedback had been applied.

### Strengths and limitations

The strengths of this study include that the interview tool had been previously used for the 2017 interviews [14] and was built on survey instruments used in previous studies [15, 16]. The semi-structured approach allowed interviewers to ask tailored questions to each interviewee given their involvement in the Partnership and ask probing questions to gain further information based on participant responses. The qualitative analysis was guided by the CFIR, which facilitated the identification and understanding of the internal and contextual factors that influenced intervention implementation and effectiveness [17]. Some limitations are noted. Although all Partnership members were invited to participate and there was a variety of strategic, implementation and research members, only 14/24 (58%) Partnership members agreed to participate and most interviewees were from organisations involved in implementation or evaluation. All food manufacturers engaged by the implementation team were also invited to participate, however only 7/19 (37%) stakeholders agreed to participate, and these interviewees were from larger companies and their experiences may not be representative of all companies engaged.

## Conclusions

The establishment of a Partnership with diverse skills and experience facilitated collaborative action and intervention delivery. Monitoring and evaluating implementation informed strategy adaptations which allowed optimisation of Partnership strategy. Future partnerships should consider the importance of developing strong communication networks, particularly between strategic and implementation-levels, interventions that fit the context and utilise available contextual mechanisms, and the balance between intervention adaptation and maintaining intervention fidelity.

## Data Availability

The datasets used and/or analysed during the current study are available from the corresponding author on reasonable request.

## Abbreviations

DALY: Disability-adjusted life year
TGI: The George Institute for Global Health
IPAN: Institute for Physical Activity and Nutrition
SGSA: State government or statutory agency
CFIR: Consolidated framework for implementation research
